# Predicting Satisfaction With Chat-Counseling at a 24/7 Chat Hotline for the Youth: Natural Language Processing Study

**DOI:** 10.2196/63701

**Published:** 2025-02-18

**Authors:** Silvan Hornstein, Ulrike Lueken, Richard Wundrack, Kevin Hilbert

**Affiliations:** 1 Department of Psychology Humboldt-Universität zu Berlin Berlin Germany; 2 German Center for Mental Health (DZPG), Partner site Berlin/Potsdam Potsdam Germany; 3 Krisenchat gGmbH Berlin Germany; 4 Department of Psychology HMU Erfurt - Health and Medical University Erfurt Erfurt Germany

**Keywords:** digital mental health, mental illness, mental disorder, adolescence, chat counseling, machine learning, artificial intelligence, large language model, natural language processing, deep learning

## Abstract

**Background:**

Chat-based counseling services are popular for the low-threshold provision of mental health support to youth. In addition, they are particularly suitable for the utilization of natural language processing (NLP) for improved provision of care.

**Objective:**

Consequently, this paper evaluates the feasibility of such a use case, namely, the NLP-based automated evaluation of satisfaction with the chat interaction. This preregistered approach could be used for evaluation and quality control procedures, as it is particularly relevant for those services.

**Methods:**

The consultations of 2609 young chatters (around 140,000 messages) and corresponding feedback were used to train and evaluate classifiers to predict whether a chat was perceived as helpful or not. On the one hand, we trained a word vectorizer in combination with an extreme gradient boosting (XGBoost) classifier, applying cross-validation and extensive hyperparameter tuning. On the other hand, we trained several transformer-based models, comparing model types, preprocessing, and over- and undersampling techniques. For both model types, we selected the best-performing approach on the training set for a final performance evaluation on the 522 users in the final test set.

**Results:**

The fine-tuned XGBoost classifier achieved an area under the receiver operating characteristic score of 0.69 (*P*<.001), as well as a Matthews correlation coefficient of 0.25 on the previously unseen test set. The selected Longformer-based model did not outperform this baseline, scoring 0.68 (*P*=.69). A Shapley additive explanations explainability approach suggested that help seekers rating a consultation as helpful commonly expressed their satisfaction already within the conversation. In contrast, the rejection of offered exercises predicted perceived unhelpfulness.

**Conclusions:**

Chat conversations include relevant information regarding the perceived quality of an interaction that can be used by NLP-based prediction approaches. However, to determine if the moderate predictive performance translates into meaningful service improvements requires randomized trials. Further, our results highlight the relevance of contrasting pretrained models with simpler baselines to avoid the implementation of unnecessarily complex models.

**Trial Registration:**

Open Science Framework SR4Q9; https://osf.io/sr4q9

## Introduction

Most mental health disorders develop early in life [1,2], causing a massive burden on an individual [3], as well as societal, level [4]. This makes early intervention in youth highly relevant [5]. In sharp contrast to the need, accessing help has been described as challenging for young people [5-7]. Therefore, low-threshold services are needed to tackle the burden of mental illness [8].

One such form of intervention gaining popularity is chat-based counseling hotlines [9-11]. Smartphones and chat interactions play a crucial role in youth life [12,13]. The ability to access help within their native digital life reduces numerous health care barriers, making the services a common first access point of help for youth [14]. Indeed, heavy utilization and adoption of those services have been reported globally [14-16]. In addition, the first evidence supports the acceptability [14] and effectiveness [17] of 24/7 chat services.

Considering the increasingly established relevance of those hotlines, the implementation of technological innovation could be highly impactful for the timely and efficient provision of care to youth. Repeatedly, artificial intelligence (AI) has been framed as a key potential for improvements in mental health care [18,19], as well as within digital settings [20]. As AI depends on the availability of large and high-dimensional datasets, chat services seem a quite promising candidate for that. This has indeed been used for diverse natural language processing (NLP) approaches, the subbranch of AI dealing with language. For example, an NLP-based triaging system has been reported to be able to reduce waiting times for those in crisis at a chat hotline [21]. Data-driven decisions regarding further treatment paths have also been investigated by looking into the prediction of recurrent chatting [22] or premature departure from conversations [23]. As suicide risk is a common case at chat hotline services [24], other work focused on early detection and intervention in those situations. Here, several model structures and algorithmic approaches have been suggested [25,26].

This study intends to contribute to the development of NLP approaches within youth chat counseling hotlines. Specifically, the promising but underinvestigated use case of automated evaluation of service quality will be explored. A recent study linked asynchronous chat counseling interactions with reported outcomes and satisfaction of the chatters, using a large dataset of more than 150,000 clients and reporting promising effect sizes of multiple *R*’s of around 0.45 [27]. Another past approach investigated the prediction of chat quality on a label of 675 transcripts of chat counseling sessions [28]. However, while we were not able to find a similar-minded approach within 24/7 hotline services, automated quality evaluation seems particularly relevant for those. Early experiences with help seeking have been linked with future help-seeking behavior in the past [29]. As often being the first contact with any kind of institutionalized help for youth [14], the satisfaction with this interaction is therefore arguably highly relevant for further help-seeking behavior. The reliable identification of those with negative experiences would allow a timely intervention by following up or referrals to other services. Second, the low threshold nature of counseling hotlines makes evaluation more difficult, as it is hard to collect follow-up responses from young help seekers. For example, the aforementioned study of chat hotline effectiveness reported a response rate of 22% among the users [17]. There is also the risk of a bias toward those more satisfied being more likely to respond, which is seen as a common methodical problem in evaluation sciences [30,31]. The ability to estimate the satisfaction with the service out of the conversation data for those who did not respond to any follow-up surveys could therefore significantly improve the evaluation and monitoring of the service quality.

In light of the relevance of the automated evaluation of chat interactions at chat hotlines, as well as the interventions raising relevance for youth mental health care, this project uses a naturalistic sample of 2609 young chatters that were counseled by the German 24/7 hotline service krisenchat. Feedback regarding the perceived helpfulness of the chat is used to train classifiers on the anonymized consultation texts. Performance is evaluated on a previously unseen test set addressing the feasibility of the approach, hypothesizing that we can significantly predict the feedback response by the chatter. Additionally, we assume that applying a pretrained transformer-based model as the state-of-the-art NLP will allow us to outperform a simpler non–transformer-based approach.

## Methods

### Preregistration

This study was preregistered at Open Science Framework [32]. The preregistration was updated once, as we adapted the used statistical test for the algorithm comparison (see the *Final Evaluation* section under *Methods*) and corrected the questionnaire item used for the outcome variable. We used the checklist for reporting machine learning studies by Klement and El Emam [33], which can be found in Multimedia Appendix 1. Due to legal restrictions regarding the highly vulnerable sample of this study, we are unable to share the dataset. However, the code used for training the algorithm and predicting the helpfulness can be found on GitHub [34], as a starting point for future work.

### Ethical Considerations

The data collected and used for this study were part of a larger research project that was ethically approved by the University of Leipzig (372/21-ek). Additionally, we submitted the proposed secondary data analysis to the ethics committee of the Humboldt-Universität zu Berlin. They confirmed that this analysis does not require additional approval. Before the use of this study, the data were subject to a multistep anonymization procedure. Specifically, personally identifying information was marked by counselors and deleted by the organization. Additionally, there also was an automatized method in place to delete names and locations that might have been missed by the counselors. Finally, a k-anonymity principle was applied, deleting all words that were not part of at least 5 different chats.

### Setting and Intervention

The anonymized data used for this study were provided by krisenchat, a German 24/7 chat counseling service for people aged up to 25 years. At krisenchat, those contacting the service through WhatsApp are provided with chat counseling, either by volunteer or employed psychologists, psychotherapists, or social workers. A central aspect of the consultations is the provision of exercises and resources, for example, by sharing YouTube videos, blog posts, or providing them within the chat. However, counselors are also trained in providing emotional support as needed, as well as providing information about mental health care structures in Germany, such as access to psychotherapy or the youth office.

### Sample

Data were accessed and shared by the organization on January 17, 2024. On this date, there were feedback questionnaires available for 4560 chatters. Those questionnaires were sent out as part of a larger research project on the service [14]. A total of 264 participants were either younger than 13 years or older than 25 years of age and therefore excluded. While the upper age limit resulted from the scope of the service, the lower age limit resulted from data privacy considerations. An additional 1631 of the chatters were in contact with the service in the last 4 months. A help seeker’s inactivity for at least 4 months is an organizational requirement for assuming the consultation purpose has ended and the chat is deleted by anonymization. Accordingly, active chats were also excluded, leading to 2664 concluded conversations and the related feedback questionnaire, with feedback provided between July 22, 2022, and September 17, 2023. For those cases, all messages exchanged between help seekers and counselors within 72 hours before the response to the feedback questionnaire were included. We then excluded cases where conversations consisted of fewer than 10 messages. This led to additional exclusions and resulted in a final sample of 2609 chatters. Their consultations consisted of 141,404 messages, 82,335 by the help seekers and 59,052 by the counselors. Therefore, on average, there were 54 messages exchanged in the three days before the feedback response, 23 messages by the counselor and 31 messages by the help seeker.

### Outcome Variable

The feedback questionnaire answered by the chatters included several questions regarding the chat interaction (see Multimedia Appendix 2 for the full questionnaire). For this study, we decided on the use of a single item asking for the helpfulness of the chat (“Did the chat help you?” in German: “Hat dir der Chat geholfen?”), as being the most direct assessment available of chat quality and success, as perceived by the young clients. While the item had four possible answers (“Yes,” “Rather Yes,” “Rather No,” and “No”), we decided to dichotomize it into “Yes” or “No.” Reasons for that were improved actionability (as most clinical decision-making is binary by nature, such as providing additional help—yes or no), as well as considering the high-class imbalance. Overall, 89% (n=2332) of the chatters rated the chat as helpful. Specifically, 61 chatters responded with “No,” 216 chatters responded with “Rather No,” 1138 chatters responded with “Rather Yes,” and 1194 chatters responded with “Yes.”

### Algorithm Training

All decisions regarding algorithmic specifications were made on the 80% of the available data used as a training set. Specifically, we separated the newest 20% of the consultations (522 chats who submitted their feedback after May 27, 2023) as a test set, a commonly used approach to mimic the evaluation of a previously implemented model (eg, [35]).

For our non–transformer-based approach, we preprocessed the data by lowering all words, deleting stop words, and using a lemmanizer [36]. Afterward, a term frequency-inverse document frequency (TF-IDF) vectorizer was used for feature extraction. This vectorizer counts the occurrences of words and weights them based on their frequency across the whole sample. This algorithm was trained using a 5-times repeated 5-fold stratified cross-validation principle. Hyperparameters were tuned using Bayesian optimization maximizing the receiver operating characteristic (ROC) area under the curve (AUC) score for 250 iterations. While there has been some discussion about the applicability of this metric facing class imbalance (eg, [37]), we saw its appropriateness backed up by systematic comparisons [38] and analysis [39] on the issue. All hyperparameters optimized during this procedure are summarized in Table 1. Those also included, as suggested by a reviewer, the range of ngrams used by the vectorizer. Therefore, bigrams and trigrams of words of the messages were also usable as predictors. The used over- or undersampling method was also selected during this procedure, comparing oversampling, undersampling, and Synthetic Minority Oversampling Technique [40]. As a classifier, we applied and tuned an extreme gradient boosting (XGBoost) [41] classifier, as well as a logistic regression. The training pipeline can be found on GitHub.

**Table 1:** Overview of shortlisted transformer-based models.

Model	Input length, n	Source
uklfr/gottbert-base	512	[42]
distilbert/distilbert-base-german-cased	512	[43]
LennartKeller/longformer-gottbert-base-8192-aw512	8192	[44]

We used hugging face for all transformer-based approaches [42]. We shortlisted GottBERT [43], as well as a German DistilBERT model [44], as language-specific models to be evaluated. However, we assumed that a significant share of our data would exceed those models’ input length. Therefore, we also intended to evaluate a Longformer model [45]. This model can process much longer input sequences at reasonable computational costs by applying a sparse attention mechanism (see Table 1 for the shortlisted models including links). We also intended to explore over- and undersampling, as well as class weights to tackle the class imbalance. To represent the chat structure appropriately to the algorithm, we introduced two new special tokens to the models, named “[USER]” and “[CNSLR].” Those were added at the beginning of each message, presenting the conversation structure in a processable format to the models. For hyperparameter tuning, a grid search across the learning rate (2×10^–5^, 3×10^–5^, and 5×10^–5^) and the batch size (1, 2, and 4) was performed for the preselected most promising model. The training and tuning were done at a stratified train-validation split (70:30 of the data used for algorithm training), as the repeated cross-validation principle applied for the TF-IDF approach was infeasible due to computational costs. Therefore, a train-validation-test split (56:24:20) was used as an evaluation principle, with the same data being kept aside as final test data for the nontransformer approach. All transformer-based models were trained on an NVIDIA GeForce RTX 3090 graphics processing unit with 24 GB video random access memory.

### Final Evaluation

The 522 newest conversations with feedback were used as a test set. The distribution of the outcome did not differ significantly between the training and test data (t_520_=–1.1; *P*=.30). We decided to predict the outcome with the best performing TF-IDF approach and the most promising transformer approach, as identified on the train set as described above. We then applied a permutation test [46] to evaluate the significance of both algorithms. Finally, we contrasted the achieved AUCs of the two approaches, applying a DeLong test [47], which has been suggested for this scenario [48]. We decided for this procedure above the 5×2 McNemar test [49] originally proposed in our preregistration. This reconsideration was mainly made due to the inability of the McNemar test to statistically compare AUC scores. The comparison of accuracies seemed disadvantageous to us, as focusing on the performance for one specific threshold. In contrast, considering the different proposed use cases, we were more interested in a threshold-independent comparison of classifier performance. As a threshold-dependent metric, we reported the Matthews correlation coefficient (MCC), which is particularly helpful in cases of imbalanced classes [50]. We followed the suggestion in the literature to use a default threshold of 0.5 [51] for the calculation of a confusion matrix and the corresponding MCC score.

### Explainability

We used Shapley additive explanation (SHAP) values [52] as an explainability framework. This game-theory–based approach is applicable for transformer models [53] and XGBoost classifier [54].

## Results

### Algorithm Training

For the TF-IDF-based approach, the best set of hyperparameters selected through the tuning approach led to a mean ROC AUC score of 0.70 (SD 0.02) across repeated cross-validation for the XGBoost classifier. For this, a minimum occurrence of the word stems for 20 different chatters and for five different counselors was selected as a hyperparameter for the vectorizers. Random oversampling was selected for handling class imbalance. Counselors word stems were only selected when occurring in 30% or less of the conversations, while chatters word stems were allowed in up to 90% of the conversations. In addition, trigrams and bigrams were included, as well as predictors (see Table 2 for all hyperparameters). This was slightly above the performance of logistic regression, scoring 0.66 for the best set of hyperparameters.

**Table 2:** Overview of tuned hyperparameters (definitions adapted from [22]).

Hyperparameters	Description	Value range	Selected parameter
max_df_chatter	Terms that appear in more chatter documents than the threshold value are ignored. The value represents the proportion of documents	0.2, 0.3, 0.4, 0.5, 0.6, 0.7, 0.8, 0.9, 1.0	0.9
min_df_chatter	Terms that appear in fewer chatter documents than the threshold value are ignored	1, 2, 5, 10, 25, 50, 75, 100, 150, 200	20
max_df_couns	Analogous to max_df_chatterfor counselor messages	0.2, 0.3, 0.4, 0.5, 0.6, 0.7, 0.8, 0.9, 1.0	0.3
min_df_couns	Analogous to min_df_chatter for counselor messages	1, 2, 5, 10, 25, 50, 75, 100, 150, 200	5
Sampling method	Method for handling imbalance	ROS^a^, RUS^b^, SMOTE^c^	RandomOverSampler
colsample_bytree	Subsample ratio of columns for growing trees	0.2, 0.4, 0.6, 0.7, 0.8, 0.9, 1.0	1.0
eta	Learning rate	0.005, 0.01, 0.05, 0.1, 0.2	0.1
gamma	Minimum loss reduction to make a further split on a leaf node	0, 0.25, 0.5, 1, 1.5, 2, 5, 10	1.5
max_depth	Maximum depth of a tree	2, 4, 6, 8, 10, 12, 14, 16	16
min_child_weight	Minimum sum of instance weight (Hessian) needed in a child	1, 5, 10, 20	10
subsample	Subsample ratio of the training instances prior to growing trees	0.2, 0.4, 0.6, 0.7, 0.8, 0.9, 1.0	0.9
use_idf	Whether to term frequencies should be reweighted by the inverse document frequencies	True, false	True
ngram_range	Length of word sequences used as predictors	(1,1), (1, 2), (1,3)	(1,3)

^a^ROS: random over sampler.

^b^RUS: random under sampler.

^c^SMOTE: Synthetic Minority Oversampling Technique.

For the transformer-based approach, we reached a ROC AUC of 0.58 for the DistilBERT and 0.59 for the GottBERT models, using class weights (9:1) and five epochs. Comparable performances were reached when random oversampling was used instead of the class weights. We expected the performance to be limited by strong truncation. Therefore, we explored the average length of the input sequence with DistilBERT as tokenizer. Data points in the train set contained on average 1889 (SD 873) tokens, showing that those models could just use a share of the available data on the chat conversations. However, with the longest conversation holding 8507 tokens, the Longformer model structure seemed capable of capturing nearly all information contained in our data. Indeed, using the Longformer model in combination with class weights (9:1), three epochs, a learning rate of 3e-5, and a batch size of one resulted in a significantly higher ROC AUC of 0.69. Neither other methods for handling class imbalance nor different epoch sizes lead to a further improved performance.

### Final Evaluation

**Figure 1:**
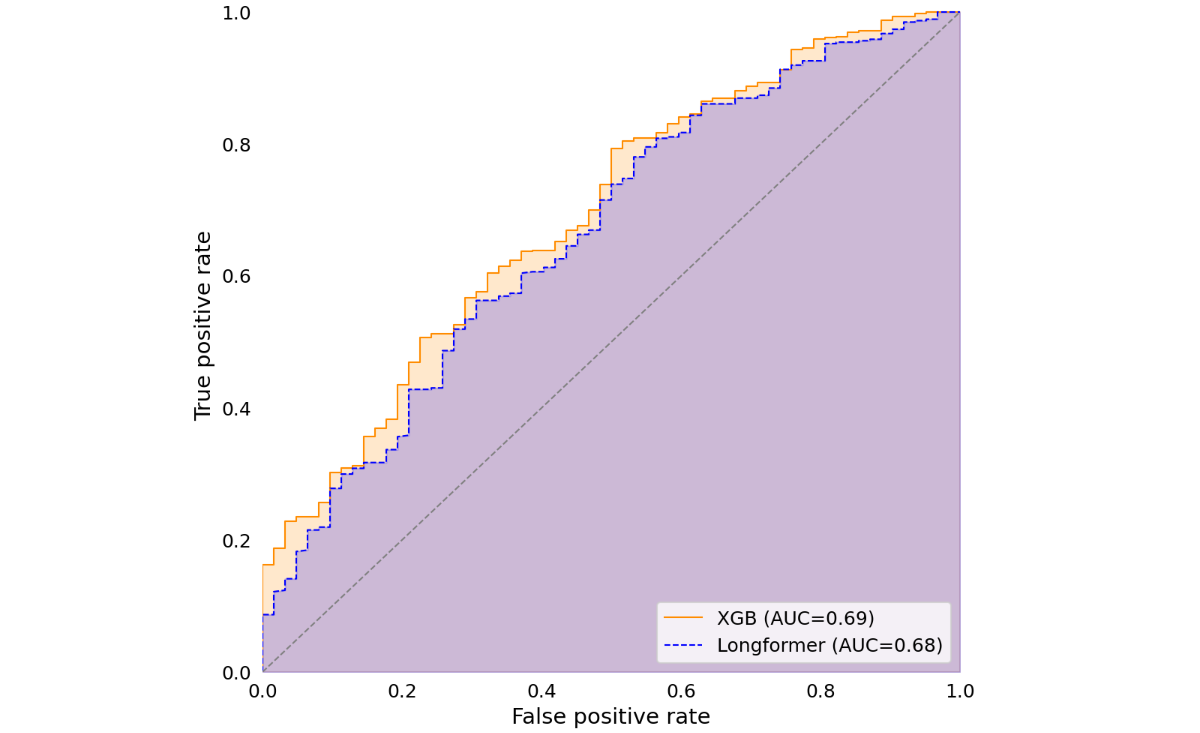
ROC AUC curves comparing the two algorithms. AUC: area under the curve; ROC: receiver operating characteristic; XGB: extreme gradient boosting.

While the performance between the transformer and non–transformer-based approach was similar during training (0.69 vs 0.70), this comparison is limited by the differences in the used validation principle. However, the large previously unseen test set allowed us the comparison of the two best-of-class models in a final evaluation. Here, we reached an ROC AUC of 0.68 for the Longformer model and an ROC AUC of 0.69 for the TF-IDF–based approach, both significantly outperforming randomness in a permutation test (*P*<.001 for both). However, as expected, considering the similar performance, there was no significant difference between the two approaches (*P*=.69). The ROC curves are plotted in Figure 1, showing how threshold and model performance interacted.

**Figure 2:**
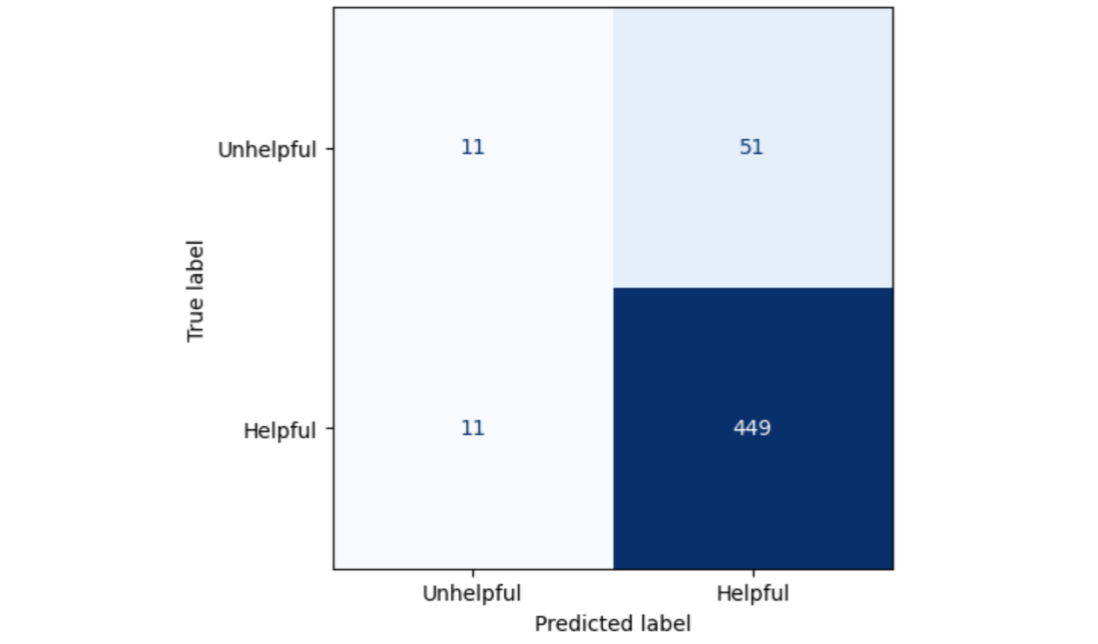
Confusion matrix for the selected threshold for the TF-IDF algorithm. TF-IDF: term frequency-inverse document frequency.

Consequently, we used the TF-IDF approach as the simpler algorithm for further insights, as well as the explainability approach. The average precision score here was 0.93 (SD 0.02) on the test set. The MCC score for the default threshold of 0.5 was 0.25 on the test set. The confusion matrix on this threshold can be found in Figure 2. Here, a positive predictive value of 0.90 and a negative predictive value (NPP) of 0.50 were achieved, with “positive” being coded as helpful. The sensitivity was 0.98 and the specificity was 0.18.

### Explainability

**Figure 3:**
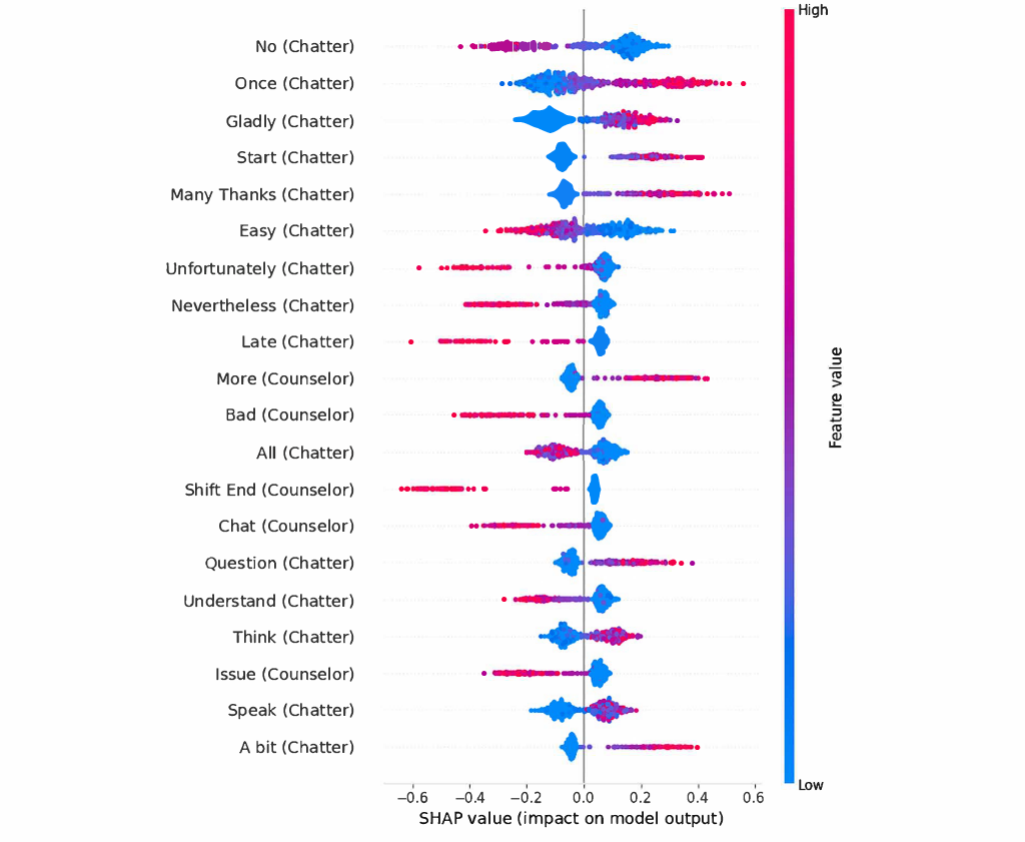
The 20 most predictive word stems as identified by the SHAP approach for the TF-IDF algorithm. SHAP: Shapley additive explanations; TF-IDF: term frequency-inverse document frequency.

We applied SHAP values on the vectorizer-based approach. The most predictive word identified here was “no” by the chatters, being associated with a higher chance of an unhelpful perceived chat. Two other predictors of unhelpfulness were the word “bad” (original: “schlimm”) by the counselor, as well as “nevertheless” (original: “trotzdem”) by the chatter, and “further on” (original: “weiterhin”) by the counselor. In addition, some bigrams were among the most predictive variables. For example, “shift end” (German: “Schicht endet”), indicating that a counselor had to end a conversation due to their shift being over, was associated with negative feedback. For an improved understanding of the context those words were used, we looked into chats using those and giving negative feedback afterward. While “no” was used in diverse settings, there was a notable number of cases where chatters denied the counselor’s offering of further help such as an exercise. “Bad” was used on several occasions where chatters reported highly traumatic experiences they had. Finally, “further on” was a phrase repeatedly used by counselors to announce the end of their shift and offer further support from a colleague afterward. There were also several words being predictive of perceived helpfulness. Several of those implied that a chatter expressed satisfaction with the interaction at the end of a chat. For example, the word stem “thanks” (original: “dank”) was predictive of higher perceived helpfulness, as was “great” (original: “toll”). We also investigated those conversations that were predicted with the highest likelihood of being labeled as unhelpful afterward. Again, there were several cases included where chatters rejected suggested exercises by the counselor. In addition, in several conversations with a high risk of unhelpfulness, it was reported that mental health care is already received, such as regularly seeing a psychiatrist or being hospitalized in a clinic. As one of the core functions of chat hotlines is the redirection into care, it might be harder to make a satisfying offer to those. The 20 most predictive words as identified by the tree-based SHAP approach can be found in Figure 3.

## Discussion

### Primary Findings

This project investigated the use of NLP techniques for an automated evaluation of the perceived helpfulness of chat-based counseling. We were able to reach a ROC AUC of 0.67 on the previously unseen test set for a transformer, as well as for a non–transformer-based approach. Our explainability part revealed several linguistic markers of perceived unhelpful chat consultations such as the written expression of thankfulness, or the extensive use of the word “no” for rejecting the different offers made by counselors.

The reached performance was moderate, though significant and in line with past work from the identical settings [22]. However, the feasibility of an AI use case always depends on the performance considering the proposed use case. The given study implied two potential uses of predicted helpfulness of the chats.

The first use case was the real-time identification of unsuccessful consultations, as perceived by the chatter. Due to the very harmful impact of such experiences, those predictions could be used for a tailored follow-up, for example, with details of different treatment options for those affected. In our example, we would have identified 30 of the 62 unhelpful rated conversations with the approach, though 79% of all identified cases would have been false negatives (with negative referring to perceived unhelpfulness).

An alternative approach would have been a much stricter threshold, letting us mark significantly less chats but with higher NPP. For example, on a threshold of 0.3, our NPP would have doubled. However, the consequences of wrongly identifying chatters as unsatisfied might be less relevant than missing those being unsatisfied in light of the possible negative consequences of further help seeking. Overall, whether one of those approaches could be valuable would depend on whether the benefits for those correctly identified are larger than the costs of providing the intervention based on the prediction. Finally, this is an empirical question that we cannot answer here sufficiently. This highlights the large need for randomized controlled trials for prediction studies, moving from feasibility to actually showing clinical benefits [55].

A second use case of the proposed algorithm lies less on the individual and more on a population-based level. As evaluation within naturalistic and low-threshold settings is commonly difficult, the developed algorithm could be applied to those who did not respond to feedback questionnaires. This application would allow a better-informed estimation of satisfaction with the service where just a minority provides active feedback. A reliable estimate of this core metric of the service would propose a huge value for organizational purposes. Without any alternative of estimating the satisfaction of those not providing feedback being available, the proposed algorithm already provides an improvement over the status quo as clearly performing above the chance level. However, particularly for systematic comparison of, for example, monthly satisfaction, the question arises whether the performance is sufficient for reliable inference. Here, simulation studies might help to better understand the relation between performance and the reliability of algorithm-based evaluation.

### Secondary Findings

Interestingly, there was no further gain in predictive capability by using the computational heavy and pretrained Longformer model. The failure of more complex NLP models to outperform simpler ones is not unique to the given setting and has been reported before [56-58]. However, based on the literature, we started the work on this paper with an opposing hypothesis. For example, a popular study [59] compared Bidirectional Encoder Representations from Transformer–based approaches with TF-IDF–based algorithms and reported a clearly better performance for the former. An in-depth look into the used methods provides several possible explanations for the diverging results. First, the cited study used a larger sample of 50,000 distinct cases, while using the much smaller Bidirectional Encoder Representations from Transformer base model. Therefore, the dataset size might have been insufficient to finetune such a sophisticated model. Second, the use case is different, while algorithmic performance is highly case specific. The cited study focuses on sentiment analysis. Arguably, the extraction from word vectors into higher-dimensional spaces like sentiment as done by transformer models is particularly relevant here. While our explainability approach revealed some sentiment-related predictors like words of thankfulness, overly sentiment seemed less central than it is for movie reviews as in the aforementioned study. Finally, it remains unclear how much the advantage of simpler models is used in comparative studies. For example, in our approach, we were able to perform extensive hyperparameter tuning using sophisticated cross-validation principles. The relevance of this to produce generalizable results, and therefore, realistic performance estimates is well established [60,61]. Such approaches are hard to reproduce at feasible computational costs for transformer-based models for a lot of ML practitioners in their day-to-day work. However, waiving those techniques also for the baseline is arguably biasing the comparison against them, as their better capability to be trained with extended cross-validation principles is a real benefit that might translate into predictive performance. Particularly, small predictive performance differences as reported regularly (eg, [25]) might disappear with decent hyperparameter tuning and cross-validation.

In conclusion, while the actual outperformance seems dependent on setting and data, the results of this study, as well as the aforementioned studies, highlight the relevance of benchmarking complex models with simpler ones. Otherwise, overly complex models might be implemented without benefits. There are numerous studies that apply interesting and promising algorithmic approaches but do not compare them with a simpler baseline at all (eg, [62-64]). However, we also argue that a fair comparison includes the utilization of hyperparameter tuning and cross-validation for computationally lighter models.

### Limitations

There were limitations to the approach in this paper. First, while we predicted the helpfulness of a chat as perceived by chatters, this perception does not equal to actually being clinically beneficial. For example, in the aforementioned study by Imel et al [27], the association between message content and satisfaction was much stronger than the association between content and symptom reduction. Therefore, future work could benefit from associating chat messages with clinically validated questionnaires as output. However, arguably changes in symptoms are difficult to measure in hotline settings, where a majority of chatters just contact the service once. Second, we were only able to train the algorithms on the data of those who responded to the feedback questionnaire. This might have introduced a bias, in case of systematic differences between those providing feedback and those who do not. Third, we focused on the application of the Longformer model in the transformer-based approach of this paper. Future work might also benefit from exploring task-specific adaptions of the used algorithms in detail. In addition, different methods of handling long text inputs such as BELT [65] might enable a better performance. Notably, there were no mental health–specific smaller models available in German. Those exist for other languages and use cases [66]. Such models, for example, pretrained on youth mental health data in German, could provide further performance gains as well. Finally, while we used a test set for a final one-time evaluation, this test set still came from the same chat counseling service. However, the relevance of truly external test sets has been highlighted repeatedly as being relevant for more valid claims regarding the generalizability of a chosen approach (eg, [67]).

### Conclusions

In summary, there is a predictive signal regarding the perceived service quality in the chat messages at a 24/7 chat hotline for youth. This opens interesting use cases in the quality control and evaluation efforts at those hotlines. Future work such as the randomized evaluation of interventions based on the predicted helpfulness is needed for moving toward real-world implementation.

## Appendix

Multimedia Appendix 1 Consolidated reporting guidelines for prognostic and diagnostic machine learning modeling studies.

Multimedia Appendix 2 Full questionnaire sent out to chatters, original (German) and English translation.

## Abbreviations

AI: artificial intelligence
AUC: area under the curve
MCC: Matthews correlation coefficient
NLP: natural language processing
NPP: negative predictive value
ROC: receiver operating characteristic
SHAP: Shapley additive explanations
TF-IDF: term frequency-inverse document frequency
XGBoost: extreme gradient boosting
