# Supplementary material for: Predicting Satisfaction With Chat-Counseling at a 24/7 Chat Hotline for the Youth: Natural Language Processing Study
Source: JMIR AI. 2025 Feb 18;4:e63701. doi: 10.2196/63701 (PMC11888103; doi:10.2196/63701)
Supplement: Multimedia Appendix 1 [file ai_v4i1e63701_app1.docx]

**Consolidated reporting guidelines for prognostic and diagnostic machine learning modeling studies**

| **#** | **Item** | **Y** | **N** | **NA** | **Location / Reasoning** |
| --- | --- | --- | --- | --- | --- |
| **Study Details** | | | | | |
| 1.1 | *The medical/clinical task of interest* | x |  |  | Introduction |
| 1.2 | *The research question* | x |  |  | Introduction |
| 1.3 | *Current medical/clinical practice* | x |  |  | Introduction |
| 1.4 | *The known predictors and confounders to what is being predicted / diagnosed* |  | X |  | We could not identify a significant body of work on predictors of chat satisfaction in 24/7 chat hotlines |
| 1.5 | *The overall study design* | x |  |  | Methods |
| 1.6 | *The medical institutional setting(s)* | x |  |  | Methods |
| 1.7 | *The target patient population* | x |  |  | Methods |
| 1.8 | *The intended use of the ML model* | x |  |  | Discussion |
| 1.9 | *Existing model performance benchmarks for this task* | x |  |  | Introduction |
| 1.10 | *Ethical and other regulatory approvals obtained* | x |  |  | Methods |
| **The Data** | | | | | |
| 2.1 | *Inclusion / exclusion criteria for the patient cohort* | x |  |  | Methods |
| 2.2 | *Methods of data collection* | x |  |  | Methods |
| 2.3 | *Bias introduced due to the method of data collection used* | x |  |  | Discussion |
| 2.4 | *Data characteristics* | x |  |  | Methods |
| 2.5 | *Methods of data transformations and preprocessing applied* | x |  |  | Methods |
| 2.6 | *Known quality issues with the data* | X |  |  | Limitations |
| 2.7 | *Sample size calculation* |  | x |  | As this was a naturalistic study, the sample size was determined by the amount of available data. |
| 2.8 | *Data Availability* | x |  |  | Methods |
| **Methodology** | | | | | |
| 3.1 | *Strategies for handling missing data* |  |  | X | No missing data |
| 3.2 | *Strategies for addressing class imbalance* | X |  |  | Methods |
| 3.3 | *Strategies for reducing dimensionality of data* | X |  |  | Methods |
| 3.4 | *Strategies for handling outliers* |  |  | X | No outliers |
| 3.5 | *Strategies for data augmentation* |  |  | X | not used |
| 3.6 | *Strategies for model pre-training* | X |  |  | Methods |
| 3.7 | *The rationale for selecting the machine learning algorithm* | X |  |  | Methods |
| 3.8 | *The method of evaluating model performance during training* | X |  |  | Methods |
| 3.9 | *The method used for hyperparameter tuning* | X |  |  | Methods |
| 3.10 | *Model’s output adjustments* | X |  |  | Results |
| **Evaluation** | | | | | |
| 4.1 | *Performance metrics used to evaluate the model* | X |  |  | Methods |
| 4.2 | *The cost or consequence of errors* | X |  |  | Discussion |
| 4.3 | *The results of internal validation* | X |  |  | Results |
| 4.4 | *The final model hyperparameters* | X |  |  | Results |
| 4.5 | *Model evaluation on an external dataset* | X |  |  | Results |
| 4.6 | *Characteristics relevant for detecting data shift and drift* | X |  |  | Limitations |
| **Explainability and Transparency** | | | | | |
| 5.1 | *The most important features and how they relate to the outcome(s)* | x |  |  | Results |
| 5.2 | *Plausibility of model outputs* | X |  |  | Results |
| 5.3 | *Interpretation of model's results by an end-user* |  |  | X | For the two proposed use cases no presentation of results to end-users would be intended |
