# Supplementary material for: Predicting Satisfaction With Chat-Counseling at a 24/7 Chat Hotline for the Youth: Natural Language Processing Study
Source: JMIR AI. 2025 Feb 18;4:e63701. doi: 10.2196/63701 (PMC11888103; doi:10.2196/63701)
Supplement: Multimedia Appendix 2 [file ai_v4i1e63701_app2.docx]

**Item 1:**

German:

Toll, dass du uns helfen willst! Deine Teilnahme ist freiwillig und dauert keine 3 Minuten. Deine Antworten helfen, krisenchat besser zu machen und zu verstehen: Warum schreiben junge Menschen krisenchat? Und wie können wir jungen Menschen helfen? Wir behandeln deine Daten streng vertraulich. Nur krisenchat und unsere universitären Partner verarbeiten deine personenbezogenen Daten. Dritte bekommen keine Daten, durch die du erkannt werden kannst. [Rechtsgrundlage für die Datenverarbeitung ist Art. 6 lit a. DSGVO.] (<https://krisenchat.de/datenschutz>) Wenn du zustimmst, bist du einverstanden, dass krisenchat und unsere universitären Partner deine personenbezogenen Daten verarbeiten.

English:

We appreciate that you want to help us! Your participation is voluntary and will take less than three minutes. Your answers help krisenchat to improve and to understand why young people write to krisenchat and how we can help them. We keep your data strictly confidential. Only krisenchat and our university partners will process your data. No-one else will have access to data that can be used to identify you. [The legal basis for data processing is Art. 6 point a. GDPR] (https://krisenchat.de/en/datenschutz).If you consent, you agree to the processing of your personal data by krisenchat and our university partners.

**Item 2:**

German:

Wie sehr haben dich die folgenden Gefühle belastet, bevor du krisenchat geschrieben hast:

1. traurig, niedergeschlagen oder ohne Lebensfreude?
2. ängstlich oder nervös?
3. gestresst?
4. wütend?
5. Gefühl, nicht die soziale Unterstützung zu bekommen, die du brauchst?

Antwortmöglichkeiten: 1- 9

English:

Before you have written krisenchat, how much have you been bothered by:

1. Feeling sad, down, or uninterested in life?
2. Feeling anxious or nervous?
3. Feeling stressed?
4. Feeling angry?
5. Not having the social support you feel you need?

Choices: 1 - 9

**Item 3:**

German:

Wie sehr haben dich die folgenden Gefühle belastet, nachdem du krisenchat geschrieben hast:

1. traurig, niedergeschlagen oder ohne Lebensfreude?
2. ängstlich oder nervös?
3. gestresst?
4. wütend?
5. Gefühl, nicht die soziale Unterstützung zu bekommen, die du brauchst?

Antwortmöglichkeiten: 1 - 9

English:

After you have written krisenchat, how much have you been bothered by:

1. Feeling sad, down, or uninterested in life?
2. Feeling anxious or nervous?
3. Feeling stressed?
4. Feeling angry?
5. Not having the social support you feel you need?

Choices: 1 - 9

**Item 4:**

German:

Hat dir der Chat geholfen?

Antwortmöglichkeiten: Ja, Eher Ja, Eher Nein, Nein

English:

Did the chat help you?

Choices: Yes, Rather Yes, Rather No, No

**Item 5:**

German:

Wir möchten wissen, wie lange krisenchat hilft. Dürfen wir dich dazu in ein paar Wochen nochmal befragen?

Antwortmöglichkeiten: Ja, Nein

English:

For how long does a chat with krisenchat help? Can we ask you again in a few weeks' time?

Choices: Yes, No

**Item 6:**

German:

Bekommst du zu dem, was ihr im Chat besprochen habt, schon woanders professionelle Hilfe?

Antwortmöglichkeiten: Ja, Nein

English:

Do you already get professional help elsewhere on what you were talking about in the chat?"

Choices: Yes, No

**Item 7:**

German:

Woher kennst du krisenchat? Wenn keine Antwort passt, kannst du auch was anderes eintippen.

Antwortmöglichkeiten: TikTok, Instagram, Youtube, Twitch, Reddit, Discord, Malteser, Freunde/Bekannte/Familie, eigene Suche (Google), aus der Presse, aus der Schule/Ausbildung/Uni, Telekom, nicht antworten, eigene Antwort

English:

How did you hear about krisenchat?If none of the answers match, just type in something else.

Choices: TikTok, Instagram, Youtube, Twitch, Reddit, Discord, Malteser, friends/acquaintances/family, internet search (Google)", from the media (TV/radio/newspaper), rom school/ college/ university, Telekom, no answer, own answer

**Item 8:**

German:

Würdest du Freunden, die Hilfe brauchen, krisenchat empfehlen?

Antwortmöglichkeiten: 0 - 100

English: Would you recommend krisenchat to friends who need help?

Choices: 0 - 100

**Item 9:**

German:

In welchem Bundesland lebst du? Wenn du woanders lebst, kannst du das auch eintippen.

Antwortmöglichkeiten:

Baden-Württemberg, Bayern, Berlin, Brandenburg, Bremen, Hamburg, Hessen, Mecklenburg-Vorpommern, Niedersachsen, Nordrhein-Westfalen, Rheinland-Pfalz, Saarland, Sachsen, Sachsen-Anhalt, Schleswig-Holstein, Thüringen, nicht antworten

English: In which federal state in Germany do you live? You can also type in a different answer if you live somewhere else.

Choices: Baden-Württemberg, Bavaria, Berlin, Brandenburg, Bremen, Hamburg, Hesse, Mecklenburg-Vorpommern, Lower Saxony, North Rhine-Westphalia, Rhineland-Palatinate, Saarland, Saxony, Saxony-Anhalt, Schleswig-Holstein, Thuringia, no answer

**Item 10:**

German: Wo lebst du?

Antwortmöglichkeiten:

in einer großen Stadt, in einer kleinen Stadt, in einer Vorstadt, in einem Dorf, nicht antworten

English

Where do you live?

Choices:

in a big city, in a small city, in a suburb, in a village, no answer

**Item 11:**

German:

Denke an die Bildung, Arbeit und das Geld, das deine Familie und du haben. Wie geht es euch im Vergleich zu den meisten anderen Menschen in Deutschland?

Antwortmöglichkeiten: besser, etwas besser, etwas schlechter, schlechter, nicht antworten

English:

Let’s think about the education, work and money that you and your family have. How is your standard of living compared to most other people in Germany?

Choices: better, somewhat better, somewhat worse, worse

**Item 12:**

German:

Wirst du in Deutschland wegen deiner Herkunft schlecht behandelt?

Choices: ja, sehr oft, ja, manchmal, nein, nicht antworten

English:

Are you treated badly in Germany because of where you come from?

Choices: yes, very often, yes, sometimes, no, no answer
